# Effects of Sociodemographic Factors on Access to and Outcomes in Congenital Heart Disease in the United States

**DOI:** 10.3390/jcdd11020067

**Published:** 2024-02-19

**Authors:** Justin Robinson, Siddhartha Sahai, Caroline Pennacchio, Betemariam Sharew, Lin Chen, Tara Karamlou

**Affiliations:** 1 Department of Thoracic and Cardiovascular Surgery, Heart, Vascular and Thoracic Institute, Cleveland Clinic, Cleveland, OH 44195, USA; robinsj51@ccf.org (J.R.); sahais@amc.edu (S.S.); 2 Case Western Reserve University School of Medicine, Cleveland, OH 44195, USA; 3 Cleveland Clinic Learner College of Medicine, Cleveland, OH 44195, USA; 4 Division of Pediatric Cardiac Surgery, Heart, Vascular and Thoracic Institute, Cleveland Clinic Children’s Hospital, 9500 Euclid Avenue, Desk M41, Cleveland, OH 44195, USA

**Keywords:** social determinants of health, disparities, congenital heart disease, equality, equity, socioeconomic, sociodemographic, outcomes

## Abstract

Congenital heart defects (CHDs) are complex conditions affecting the heart and/or great vessels that are present at birth. These defects occur in approximately 9 in every 1000 live births. From diagnosis to intervention, care has dramatically improved over the last several decades. Patients with CHDs are now living well into adulthood. However, there are factors that have been associated with poor outcomes across the lifespan of these patients. These factors include sociodemographic and socioeconomic positions. This commentary examined the disparities and solutions within the evolution of CHD care in the United States.

## 1. Introduction

Congenital heart defects (CHDs) are complex conditions affecting the heart and/or great vessels that are present at birth. These defects affect millions of newborns each year. The complexity of the defect(s), though, is not solely defined by the structural abnormality, but is also closely intertwined with sociodemographic and socioeconomic factors. Life expectancy has increased with advances in technology and a better understanding and care of these patients [1] and, therefore, attention has shifted to non-clinical factors that, too, have been linked to outcomes. Some of these factors that have been heavily studied in recent years are social determinants of health (SDoH) and health-related inequities, which exist in a framework of population, community, and individual levels. Systemic factors are also closely linked to inequities in both the access to and delivery of the highest quality care [2]. This commentary examines the interplay between these hierarchical SDoH and the way they impact the access to and delivery of the highest quality care, as well as current and future insights into paradigms exemplifying ways in which we have sought to reduce disparities in the care for CHDs and adult congenital heart disease (ACHD) patients in the United States (U.S.).

## 2. Definitions and Context

### 2.1. Social Determinants of Health

SDoH are the conditions in which people are born and live that are shaped by the distribution of money and resources. These determinants of health have been associated with adverse outcomes for fetuses, children, and adults with congenital heart disease [3]. In working to mitigate these disparities, understanding the systemic inequities, inequalities, and individual and institutional biases concerning racial or ethnic groups has been prioritized, as such an understanding is required in order to address these issues. [2].

### 2.2. Disparity

Disparity, as defined by Merriam-Webster, is a noticeable and usually significant difference or dissimilarity. Disparities in CHDs have been largely examined under the lens of the workforce, access to care, race/ethnicity, sociodemographic status, diagnosis, morbidity and mortality, and long-term outcomes (Table 1).

## 3. Unraveling Disparities in CHDs

### 3.1. Maternal Health and Associated Factors

Maternal health, often examined in an isolated manner, is the pinnacle at which disparities gather momentum in CHDs. These disparities start with maternal health and lived experiences that often undermine children with CHDs well before they are born. Extensive literature has documented the impact of maternal health factors, environment, and education on the outcomes associated with CHDs [4,5,6]. These studies have, time and again, highlighted the profound implications these factors have for both the mother and the fetus.

Nutritional need is one very basic factor. Women desiring to become pregnant and pregnant mothers need to have access to and consume high-quality foods, as well as prenatal vitamins. Unfortunately, disparities are ever present when it comes to the access of safe and high-quality food sources. Reports from the U.S. Department of Agriculture (USDA) revealed that approximately 12.8% (17.0 million) of U.S. households experienced food insecurity in 2022, while 5.1% (6.8 million) had very low food security [7]; among peripartum women approximately 11% experienced suboptimal food security, while almost 5% experienced low or very low food security [8]. Across socioeconomic lines and divides, for example in urban or rural communities, food deserts exist leading to insufficient nutritional options for mothers, increased rates of obesity, poor diet, and gestational diabetes, among other health issues. Notwithstanding, maternal nutritional choices can be influenced by societal and cultural norms and, again, may be limited by economics and proximity or access to food, as previously stated [6]. As one study demonstrated, these so-called “social-environmental” characteristics that affect nutrition are “…known risk factors for maternal health conditions such as diabetes and obesity [6], ” both of which are linked to the development of CHDs among fetuses and worse outcomes for both fetus and mother. Obesity and diabetes have been linked to adverse pregnancy outcomes, neonatal complications, and morbidity such as still birth, macrosomia, congenital malformations, and CHDs [9].

Moreover, a lower socioeconomic position (SEP) is a proxy for certain environmental and behavioral factors that can also be linked to adverse maternal health [10]. These factors may include smoking, drug use, alcohol use/abuse, and disadvantaged environmental living conditions [10]. Additionally, mothers with a lower SEP may be plagued by poverty, which will increase psychological stress and mental health, potentially impacting the emotional and parental needs of a child with CHDs [10]. In a systematic review and meta-analysis examining the association between maternal factors and risk of CHDs, maternal smoking, exposure to organic solvents, and diabetes were associated with a 1.16, 1.82, and 2.65 odds ratio, respectively for CHD risk in offspring. Furthermore, there is increasing knowledge that environmental pollution such as carbon monoxide and nitric oxide can impact fetal development and lead to CHDs [11,12,13].

Disparities in maternal education also exhibit a negative effect on CHD outcomes. This has been elucidated in one population-based study in California where the authors found that a maternal age <18 years, maternal education of less than 12 years, and public insurance status were associated with increased odds of a poor outcome for a child born with CHDs [5]. Determining that inequities in critical maternal factors exist is just the beginning in reducing/solving them. Unfortunately, these disparities are often compounded, with the maternal factors directly impacting and increasing the disparities within the prenatal period.

### 3.2. Prenatal Period

The prenatal period is a crucial time for the developing fetus to receive the necessary care for optimal health. However, during this time, healthcare disparities and SDoH impact the quality of and access to care. These inequities may be apparent with respect to insurance type, which can be a direct reflection of employment status and income. It has been noted that the prenatal diagnosis of CHDs is less likely among women with public insurance compared with those with private insurance [2,14]. Prenatal diagnosis of CHDs in minority or disadvantaged groups has remained a challenge. Among Black children, for example, pre-hospital diagnosis has been disproportionately lower than non-minority racial groups. Black children are more likely to be diagnosed with CHDs during an emergency department (ED) visit for other reasons [15]. Further emphasizing this point, several other studies have found that Hispanic ethnicity, lower SEP, and rural or impoverished residency were associated with decreased prenatal diagnosis even in places with universal health insurance [16,17,18,19].

### 3.3. Infancy and Early Childhood

Most notably, health insurance, again, appears to be a prominent factor that accounts for disparate outcomes, as does access to high quality care. Uninsured children have a 2-to-3-times higher mortality risk after surgical repair than their insured peers and this was further delineated across race and ethnicity whereby 11% of Asian infants, 16% of White infants, 17% of Black infants, 29.5% of Native American infants, and 29% of mixed-race infants were not insured [20]. One study found that non-risk-adjusted LOSs were longer among patients in their Medicaid cohort [21]. Additionally, neonates insured under government-sponsored insurance, or those with parents earning in the first income quartile, experienced a longer LOS compared to those with parents earning in the fourth income quartile [22]. Studies have also demonstrated that a greater distance between birth location and a cardiac surgical center carries an increased risk of mortality, with some of the literature suggesting a close to 20–28% increased risk of mortality [23,24].

Even as differences in insurance potentiate disparities in CHDs, neurocognitive development has also been determined as a critically related factor. Unfortunately, poor neurodevelopment has been seen as an outcome in children with CHDs and has been associated with lower academic achievement and speech and behavioral problems [20]. This was delineated further in the landmark Boston Circulatory Arrest Study, in which they longitudinally studied CHD patients and found that patients with transposition of the great arteries (TGA) were below the population norms and below mean average scores when compared to healthy controls in areas such as academic achievement and social cognition [25]. These issues affect the child and can manifest in areas such as repeated school absences, lower IQs, perceptual reasoning, working memory, visual perception, and executive and motor functioning skills [26].

Current American Academy of Pediatrics (AAP) and American Heart Association (AHA) guidelines recommend universal screening and long-term surveillance for neurodevelopmental disability in all children with CHDs [27]. The Congenital Heart Disease Intervention Program Trial is one such neurodevelopmental program that examines the influence of family factors on neurodevelopment and aims to provide psychoeducation, narrative therapy, problem-solving techniques, and parenting skills training, delivered in six, 1–2 h sessions by a clinical psychologist and pediatric cardiac nurse [28]. The trial found that the intervention group had significantly higher mental development scores than the control group at the six month follow-up [28]. Although this is a promising strategy, several limitations exist including the need for longer-term data. The intervention was conducted in the UK and there was no mention of race/ethnicity or any other social determinants of health factors, thereby limiting the ability to translate these results. This, like other interventional programs, is a starting point, but it should be taken into consideration that often continued care may be hampered by parental ability to access these resources, rather it may be with difficulty with in-person or virtual visits, adequate means of transportation, or access to technology can be limited [26]. Even with the view that early and longitudinal intervention may assist neurodevelopment in children, a limited number of centers offer both cardiac and neurodevelopmental rehab, or other follow-up services that may not be easily accessible locally, such as in rural communities [20]. There has been an association between living >200 miles from a center that offers neurodevelopmental evaluations and non-attendance with appointments, which is often the case for travel distance for individuals living in rural communities, thus further widening the gap between quality care and outcomes [29]. Other characteristics for non-attendance to neurodevelopmental follow-up clinics were median income below the 25th percentile, less likely to have private insurance, and less likely to be college graduates [29].

Postoperative outcomes and mortality are among the most widely studied outcomes in CHD disparities research. Notably, many studies on this topic conclude that disparities in postoperative outcomes along sociodemographic lines are not adequately explained solely by the differences in access to care, suggesting that sociodemographic factors may be an independent risk factor in determining postoperative outcomes. Risk models for postoperative outcomes often fail to account for sociodemographic factors, which may be just as important in determining survival and quality of life. Children of racial/ethnic minorities are consistently found to have increased mortality, with non-Hispanic (NH) Black children experiencing the greatest risk of mortality [30]. Similarly, another study reported an increased relative risk (RR) of death among NH Blacks and Hispanics compared to NH Whites, even after adjusting for access to care [31]. Black patients were found to have more risk factors for failure to rescue (FTR)—defined as mortality after a hospital complication—compared to NH White patients [32,33]. NH Black children were found to be at an increased risk of early childhood mortality for congenital defects such as atrial septal defects (ASDs), ventricular septal defects (VSDs), pulmonary valve atresia without VSDs, and tetralogy of Fallot (TOF) [34]. Oster and colleagues posit that there may be several factors that contribute to the racial/ethnic disparities in postoperative mortality, even when access to care is accounted for, including race/ethnicity-based differences in referral patterns, race/ethnicity of the provider, unknown prenatal exposures that vary based on race/ethnicity, and possibly actual biological differences among racial/ethnic groups [31].

The transplant waitlist mortality among racial groups is another area that has been reviewed extensively. A study found that Black children were more likely to experience waitlist mortality compared to NH White children [35]. Similarly, it was found that waitlist mortality was worse for non-White children following the 2016 revision to the US Pediatric Heart Allocation Policy (PHAP), while no significant difference was found between White and non-White waitlist mortality pre-2016 [36]. The authors posit a reason for this may be that the PHAP revision downgraded the listing status of cardiomyopathy patients, of which a majority are non-White. However, it is essential to recognize that implicit physician bias and perceived discrimination exist and continue to be a factor in healthcare decision-making. Not only is it physician bias that continues to be a factor, but also biases and misperceptions among the entire healthcare team. This may result in certain racial populations receiving unequal prioritization on transplant lists or requiring a greater disease severity threshold to be listed for transplant in the first place. Even after transplantation, Black children were more likely to have acute rejection episodes within the first three years of transplant compared to Caucasian and Hispanic children [35]. It is unknown whether there is a biological predisposition to developing rejection seen predominantly in the Black population or whether the increased incidence of rejection is due to a reason beyond genetic or physiological differences.

### 3.4. ACHD and Transition of Care

Despite the highly evolved healthcare systems in the U.S. and other first-world countries, ACHD care is fragmented worldwide, with published reports from all over the globe underscoring the need for solutions to the growing ACHD crisis [37]. Only 308 U.S. physicians have been certified in ACHD care, and based on a 2010 estimated ACHD population of 1.4 million, the expected ratio of board-certified ACHD physicians to ACHD patients in the U.S. is ~1:4500 [37].

The geographic landscape of CHD centers and professionals in the U.S. often puts those living in rural or smaller communities at a disadvantage. Only 51 ACHA ACHD Accredited Programs exist in 26 states [38]. Many of these specialized centers and professionals are found in large, urban, densely populated settings or coastal regions. To further illustrate the geographical/access disparities in adequate care, the HEART-ACHD (The Health, Education, and Access Research Trial) national multicenter study showed that patients living in the Mountain West and Pacific Northwest, for example, were more likely to have patients who had gaps in care [39]. What is more, approximately 45% of the U.S. population lives a considerable distance from these centers (i.e., a drive-time of >1 h) [37]. These patients who have prolonged drive-times are more likely to be uninsured, live below the federal poverty level, have household incomes below the federal poverty level, and are less likely to have graduated from college [37], further emphasizing the disparities with transition of care and care of adult CHD patients. Because of this disparity in the geographical location of high-quality CHD centers, the distance to a center has direct and deleterious effects on morbidity and mortality [2,40].

In transitioning from pediatric to adult level congenital care, approximately 40% of patients with congenital heart disease experience a care gap related to access [39]. Reasons for this are multifactorial and can be related to existing knowledge gaps and inadequate patient education that repaired CHDs does not mean cured CHDs. In adulthood, patients are now responsible for their own care instead of their parents, who may have understood the importance of follow-up and care during childhood. When transitioning care, many adult congenital heart disease (ACHD) patients report that the most common reason for gaps in care is a lack of knowledge regarding the importance of follow-up [2]. Affordability, and related factors such as having insurance or insurance covered by specialty providers are other issues that highlight the barriers to the access to healthcare often faced by disadvantaged populations. For example, among neonates, the use of government-sponsored insurance was associated with higher mortality as compared to patients insured by private insurance [22]. Another study similarly found that non-risk adjusted mortality was higher among patients in New York State with Medicaid [21].

Impaired neurocognitive development, common among ACHD survivors, also may limit these patients’ employability, which extends to the ability to afford and access high-quality care. Impaired neurocognitive or psychosocial issues among ACHD has a profound impact on the maintenance of care, medication adherence, and overall quality of life [41]. Although some neurodevelopmental programs exist, very few of these are offered to adolescents and adults [20]. Many ACHD patients face psychological problems such as depression and PTSD, and yet adequate psychological treatment and patient education are scarce [20]. More concerning is that psychological problems are often, and unfortunately, underdiagnosed and undertreated [42] among non-White patients.

As the ACHD population continues to rapidly expand, the need for access to quality of care will only increase. We must remember that, as outlined by the AHA accreditation statement “These adult patients do not have the same health care delivery systems in place afforded to both children with CHDs and adults with acquired heart disease. Adult CHD (ACHD) patients fall victim to increased morbidity and increased early mortality.” We should then aim to expand access for lifelong CHD care, develop models for improved portability of insurance benefits across state lines, increase attention in rural contexts and at-risk urban communities for efforts focusing on awareness, prevention, expansion of emergency management, and follow-up care, and provide lifelong insurance for patients with CHDs [20].

## 4. Framework of CHD Care

### 4.1. Regionalization and Access

Regionalization of congenital heart programs has been a topic of discussion for some time. Studies have examined this in depth and have discussed it in the context of outcomes, especially in lower income, disadvantaged populations. To that extent, Karamlou et al. noted that consolidating programs decreased the national mortality rate and increased collaboration among the local centers [43]. Other studies have noted that regionalization has been shown to reduce the variation in clinical practice and significant variation in clinical practice may be associated with adverse outcomes [43]. Often described as a limitation to regionalization is that of access to these specialized centers. Although this is a reasonable and concerning factor to consider, most CHS centers in the U.S. are located within 25 miles of one another [43]. Additionally, patients tend to travel longer distances to seek care at what they may perceive as a high-volume center of excellence [44]. Moreover, some contemporary healthcare system models have incorporated aspects of regionalization into their infrastructure, with favorable results. These models have been designed as satellite systems in which one or more small hospitals are affiliated with a large hospital —the satellite or spoke-and-hub model [43]. One perceived benefit of this model is that some patients can be treated close to home, reducing the burden on their families [43]. Not only does regionalization benefit patients, but it also benefits the system by increasing surgeon and center volume and decreasing healthcare spending [43].

Access to care, as can be defined in different domains, most importantly is that of having access to specialized cardiovascular care and, within these care systems, access to educational resources and resources for patients with cognitive and psychosocial impairment. Access to care also embodies access to a diverse group of congenital providers and access to translational services for non-English speaking patients [20,45].

Access to care, when critically scrutinized, encompasses more than regionalization’s effects on care and outcomes. Rather, individual factors such as transportation, reliability, and access to technology (i.e., internet and phone service), and time constraints, among many other aspects embody the difficulties facing socially disadvantaged populations. Moreover, inadequate access to care occurs over lifetimes, even before the pregnancy with maternal health and well-being, to the actual prenatal period before birth, and extends well beyond birth and into adult life.

### 4.2. Refugees and Asylum Seekers

Refugee children and asylum seekers within the U.S. are vulnerable populations that, within the framework of CHD care, are, too, affected by disparate healthcare conditions. There were approximately 287,129 refugees in the U.S. in 2017, with 50% of refugees being children [46]. As war and other destructive conditions surmount and continue to plague developing countries, this number is expected to grow rapidly. Moreover, many of these children have not received adequate health care before coming to the U.S. In fact, in one population-based study’s examination of a clinic that provides patient care services to immigrant/resettled refugee children, it was found that of the 366 immigrant/refugee patients, with a median age of first evaluation of 6.3 years (range 0.02–18.2 years), it was found that over 60% of the patients were newly diagnosed with simple or complex congenital heart disease [46]. Unfortunately, many of these patients lack adequate access to care beyond their diagnoses. A sobering reality is that the burden is placed solely on the patient or caretaker. However, the issues that are faced extend beyond a lack of access to comprehensive healthcare services but also include the ability to obtain insurance, limited income/unemployment which lead to poverty, lack of transportation, lack of understanding, language barriers, lower English language proficiency (which has been related to worse health,) cultural differences, problems navigating the complex U.S. healthcare system (which is often linked to unfamiliarity with the healthcare system), and traditional cultural beliefs and preferences for alternative treatments not offered or practiced in most U.S. healthcare systems [46,47,48]. 

Like many native U.S.-born CHD and ACHD patients, refugees and asylum seekers often face high levels of mental health issues related to post-traumatic stress disorder, depression, and resettlement stress. Many of the mental health issues are related to unfamiliar jobs and educational systems, lack of social support and discrimination, low educational status, lack of religious and community engagement, and loss of family members/friends, among others [49]. 

## 5. Current Tools, Predictive Modeling, and Changing the Landscape for the Future

The most validated model for calculating socioeconomic determinants of child health and development is the childhood opportunity index (COI), which was updated in 2020 to the COI 2.0. The COI 2.0 uses 29 indicators of childhood opportunity across three domains (education, health and environment, and socioeconomic), including traditional (e.g., median household income) as well as novel (e.g., access to green space) indicators to quantify barriers to quality healthcare in U.S. neighborhoods [50]. The COI has been used to calculate morbidity risk in children with congenital heart disease with high reliability. In a study of 6247 children who underwent surgery for CHDs between 2010 and 2020, a lower COI was associated with an increased early postoperative mortality and longer length of stay [51]. Another study of 6133 patients found that children with a low COI had a significantly greater adjusted risk of late death or transplant and reintervention [52]. A multicenter study found children in the lowest COI quintile were at a greater risk for post-operative mortality [50]. Given the strong association between COI and CHD outcomes, it is a valuable tool for researchers and clinicians to assess children with significant socioeconomic barriers. Recognizing these barriers should then prompt clinicians to intervene at an early stage and provide adequate resources. Identifying populations and individuals with more significant socioeconomic barriers to health is the first step in the democratization of CHD care on a community-based level as well as an individualized one. Targeted investment in low COI neighborhoods may improve post-operative outcomes. It is important to note that studies have shown that COI may not account for all racial and ethnic disparities in CHD outcomes, but it is a start [50].

**Figure 1:**
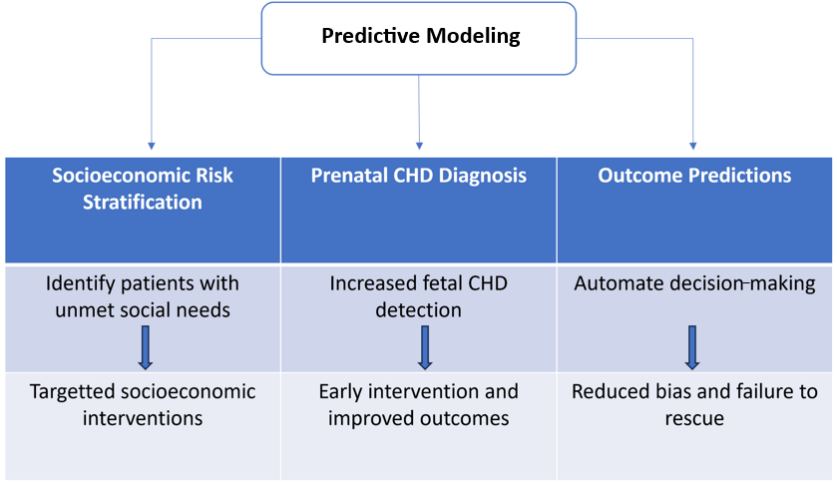
Application of predictive models in addressing congenital heart disease health disparities.

Predictive models can be used to automate decision-making and potentially reduce bias [53,54] (Figure 1). Lau and colleagues examined how automated decision-making affected the utilization of suitable venous thromboembolism (VTE) prevention upon hospital admission. They observed that introducing compulsory computerized clinical decision support led to a notable reduction in the disparity of VTE prophylaxis between Black and White patients [53]. A notable cause of disparities in CHDs is failure to rescue [33]. With the use of an automated model for predicting post-operative complications, this may reduce the notable disparity in failure to rescue [55]. For CHDs, some models predict outcomes such as in-hospital mortality, postoperative complications, postsurgical bleeding, prolonged reliance on mechanical ventilation, and hospital length of stay [56]. Machine learning (ML) and deep learning (DL) models have demonstrated their ability to predict and calculate individualized risks with high accuracy. Specifically, the Pediatric Heart Network Single Ventricle Reconstruction trial dataset has been employed to predict outcomes like mortality or the necessity for cardiac transplantation, which holds the potential for informing both clinical and organizational decision-making processes [57].

Although the effectiveness of predictive algorithms in enhancing healthcare has been demonstrated through their accurate prediction of patient deterioration based on retrospective analysis of past data, their implementation within hospital environments has yet to consistently result in enhanced patient care [17]. Achieving improvements in outcomes will demand a more comprehensive integration of these models into patient care. The current task for institutions is to determine the optimal approach for ensuring the widespread adoption of these models in patient care [17,58]. Additionally, the deployment of equitable models that can account for the risk introduced by disparities and have targeted individualized solutions that are culturally sensitive should also be integrated.

Another role of predictive models in reducing health disparities in CHDs is in prenatal screening. Prenatal detection of CHDs allows for stratification of resources and planning delivery at a surgical center with the capacity to care for high-risk infants. This, in turn, reduces preoperative complications and can improve mortality [59,60]. A cohort study of 535 neonates at the Children’s Hospital of Wisconsin found that while the prenatal diagnostic rate is improving, it remains low for those living in higher poverty or lower population density communities [60]. Improving prenatal diagnosis rates requires improved diagnostics and the identification of high-risk mothers who can benefit from screening. Models for acquiring standard cardiac imaging planes and in utero detection can assist less-experienced clinicians and operators in identifying abnormalities during the evaluation of fetal echocardiograms [56,60]. This could enhance the community’s ability to detect congenital heart defects (CHDs) at higher rates. Models to predict the likeliness of CHDs in pregnant mothers have also been growing; however, these have not been validated [59].

Beyond the utilization of predictive models and machine-learning tools to better understand and combat disparities in CHD/ACHD care, emphasis should also be placed on the research of these disadvantaged groups, which have primarily been understudied [61,62]. This scarcity of research in minority groups is apparent, with one study showing that in 255,000 randomized clinical trials (RCTs) published over the past 25 years, only 4% had minority inclusion [63]. This explains the lack of information about ethnicity and health, and the applicability of evidence generated from clinical trials to minority groups, and, in turn, decreases access to interventions and relative undertreatment [62]. Notwithstanding, some reasons for which there is a paucity of information regarding minority groups include lack of opportunity, medical ineligibility, circumstantial reasons, lack of relevant cultural understandings on the part of the researchers, and distrust of health research [62]. Although these issues are pervasive, strategies are being implanted to counteract them. One such approach is the AllofUS Research Program sponsored by the NIH, which aims to build a diverse database that can inform thousands of studies on various health conditions by recruiting a diverse participant pool. This will create more opportunities to understand disease risk factors, understand which treatments work best for people of different backgrounds (i.e., precision medicine), and connect people with the right clinical studies for their needs. Other areas that have been focused on are:

1. Access to high-speed technology and clinical databases that have increased the share of emerging disparities research [2,64].
2. Qualitative research methodologies that allow researchers to hear from populations about problems and potential solutions that may be helpful in the assessment of implicit bias or microaggressions that may be difficult to measure quantitatively [2,64].
3. Community-engaged research, which increases awareness and educates potential participants, provides education and resources about clinical research, describes methods used to protect research participants, and demonstrates researcher’s commitment to the community through ongoing engagement, therein building trust [2,64].

**Figure 2:**
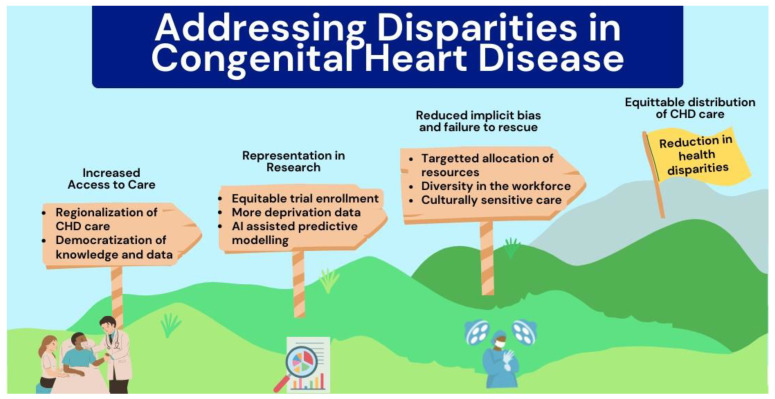
Concerted steps for addressing disparities in congenital heart disease.

Additionally, utilizing resources such as the Research Literacy Support (RLS) tool, which is an interactive tool that uses plain language principles in order to address the communication barriers regarding the purpose and process of medical research between researchers and potential participants that may impede participation [65]. Effectively addressing disparities will require a concerted effort with emphasis on increasing access to care, representation in research and among providers, as well as providing an equitable distribution of care (Figure 2).

## 6. Conclusions

Disparities in access to care and the resulting outcomes in those with CHDs in the U.S. is a complex issue that has been the focus of many studies. Disparities encompass many factors and can be explained across broad, interacting spheres that include population, systems, and individual factors. Population-level factors include access to care, systemic-level factors include reduced diversity in the CHD workforce, and individual-level factors include living conditions and genetics. Further, developing prediction models to stratify and identify at-risk populations has been an ongoing effort. The early intervention of these at-risk populations may reduce disparities, but only after there is widespread recognition that these inequities are as critical as other traditional patient factors. At the population level, proposals have included interventions surrounding institutional priorities and policies, including metrics that stratify centers based on case complexity, bolstering of partnerships between stratified centers, evaluating referral patterns and access to centers serving high-complexity patients, increasing access to cardiac surveillance programs, and increasing transparency of center outcomes [2]. Although there is heightened awareness regarding the influence of SDoH, we hope that this will translate into developing solutions and propose policy change to promote improved patient outcomes.

## Figures and Tables

**Table 1:** Sociodemographic factors affecting CHD care across time periods and populations.

Time Period and Population Affected	Sociodemographic Factors
Maternal	Insurance status Nutrition and access to high-quality food Living environment Access to high-quality care Education level Support system and social construct
Prenatal Period	Insurance status Nutrition and access to high-quality food Living environment Access to high-quality care Prenatal diagnosis
Infancy and Early Childhood	Insurance status Living environment Access to high-quality care Postoperative outcomes and mortality
ACHD and Transition of Care	Insurance status Living environment Transportation Affordability Access to high-quality care Knowledge gaps Educational level Limited ACHD providers
Refugees and Asylum seekers	Insurance status Living environment Transportation Affordability Access to high-quality care Knowledge gaps Educational level Limited income/unemployment Lack of understanding Language barriers Religious differences Cultural differences Alternative treatment choices

## Data Availability

No new data were created or analyzed in this study. Data sharing is not applicable to this article.
